# The functional antagonist of sphingosine-1-phosphate, FTY720, impairs gut barrier function

**DOI:** 10.3389/fphar.2024.1407228

**Published:** 2024-08-19

**Authors:** Sohini Sikdar, Debmalya Mitra, Oishika Das, Moumita Bhaumik, Shanta Dutta

**Affiliations:** ^1^ Division of Immunology , ICMR-National Institute for Research in Bacterial Infections (NIRBI), Kolkata, India; ^2^ Center of Radiological Research, Columbia University Irving Medical Center, New York, NY, United States

**Keywords:** FTY720, sphingosine-1-phosphate, occludin, claudin-4, gut permeability

## Abstract

FTY720 or fingolimod is a known functional antagonist of sphingosine-1-phosphate (S1P), and it is effective in treating multiple sclerosis and preventing inflammatory bowel disease (IBD). Evidence shows that its use in mice can increase the susceptibility to mucosal infections. Despite the significant contribution of S1P to barrier function, the effect of the administration of FTY720 on the mucosal barrier has never been investigated. In this study, we looked into how FTY720 therapy affected the function of the gut barrier susceptibility. Administration of FTY720 to C57BL/6 mice enhances the claudin-2 expression and reduces the expression of claudin-4 and occludin, as studied by qPCR, Western blot, and immunofluorescence. FTY720 inhibits the Akt–mTOR pathway to decrease occludin and claudin-4 expression and increase claudin-2 expression. FTY720 treatment induced increased colonic inflammation, with notably greater immune cell infiltration, colon histopathology, and increased production of TNF-α, IFN-γ, CXCL-1, and CXCL-2 than that in control mice. Taking into account the close association of “the leaky gut” and gut dysbiosis among the major diseases, we therefore can infer that the vigilance of gut pathology should be maintained, where FTY720 is used as a treatment option.

## Introduction

The mammalian gut is a multilayer system with an immunological barrier within and a physical barrier outside, thus enabling the gut microbiome to maintain equilibrium within the body. The gut barrier that maintains these luminal compounds needs to be considered. Bioactive phospholipid sphingosine-1-phosphate (S1P) has pleiotropic properties, which help in cell–cell integration (Santacreu et al., 2019), epithelial barrier regulation (Weigel et al., 2023), cell proliferation (Chen et al., 2017), migration (Chen et al. (2017) and Wang et al. (1999)), and survival (Van Brocklyn and Williams, 2012). S1P is ubiquitous in cells (Maceyka and Spiegel (2014), Le Stunff et al. (2004), and Schmidt et al. (2019)) and produced by key enzyme sphingosine kinase (SPHK) (Imamura et al. (2001) and Pulkoski-Gross et al. (2015)). SPHK catalyzes the ATP-dependent phosphorylation of sphingosine (Sph) and exists in two isoforms: SPHK1 and SPHK2 (Hatoum et al. (2017), Alemany et al. (2007), and Liu et al. (2000)). S1P is reported to play an important role in tolerance by regulating innate immunity by acting through one or more than one of its five known receptors (S1PR1–S1PR5) (Chun et al., 2002), which are G-protein-coupled receptors (GPCRs) (Goetzl et al. (2000), and Hla et al. (2001)). FTY720 or fingolimod inhibits lymphocyte egress from the thymus, spleen, and lymph nodes (LNs) into the bloodstream and lymphatic system by modulating S1P signaling, thus restricting lymphocyte trafficking to target tissues (Chiba, 2005). When FTY720 enters the body, it is phosphorylated (FTY720-P) and binds to S1PR (Wang et al., 1999; Van Brocklyn and Williams, 2012; Chen et al., 2017; Santacreu et al., 2019; Weigel et al., 2023) and internalizes the receptors, except S1PR3, preventing its downstream signaling (Sykes et al., 2014). Interestingly, FTY720-P can only activate S1PR3, which is mostly present in neutrophils, monocytes, macrophages, and B cells (Bryan and Del Poeta, 2018), inducing further downward signaling (Sensken et al., 2008). Additionally, it influences the migration of dendritic cells (DCs) (Han et al., 2015), modulates DC-mediated pro-inflammatory signaling (Zeng et al., 2012), and is a potent suppressor of regulatory T-cell (Treg) proliferation (Wolf et al., 2009). Whether FTY720 regulates lymphocyte recirculation *in vivo* in an agonistic or a functional antagonistic manner, or in both, is still up for debate (Blanc et al., 2015). The FDA has authorized the medication FTY720 (trade name Gilenya) to treat multiple sclerosis relapse (Sharma et al., 2011). Several reports showed the protective efficacy of FTY720 in various preclinical models like oxazolone (Daniel et al., 2007a), TNBS (Daniel et al., 2007b), and DSS-induced murine models of colitis (Deguchi et al., 2006), as well as IL-10-deficient mouse model (Mizushima et al., 2004). FTY720 is in clinical trial for inflammatory bowel disease (IBD) patients (Danese et al., 2018). Furthermore, it showed therapeutic efficacy in the treatment of graft-versus-host disease (Ryu et al. (2020) and Gauthier et al. (2018)) and rheumatoid arthritis (Tsunemi et al. (2010) and Nakano et al. (2022)) in mouse and viral infection models (Miyamoto et al., 2001). FTY720 prevents colitis by modulating the colitogenic CD4^+^T cells from the bone marrow (Fujii et al., 2008). However, being a structural homolog of S1P, which helps in promoting barrier function, the effect of FTY720 on the mucosal barrier has never been elucidated.

The body’s largest interaction with the outside world is the gastrointestinal epithelium, which effectively creates a barrier that limits the mucosa’s ability to absorb luminal toxins and antigens while permitting nutrient and water absorption. Epithelial cells use both the paracellular and transcellular transport pathways to form this specific barrier (Laksitorini et al., 2014). The paracellular route, however, which is controlled by the tight junction (TJ), is in charge of the highest level of apical cell–cell adhesion and has drawn the most attention for its function in controlling mucosal permeability in both healthy and pathological settings (Paradis et al., 2021). The TJ (also referred to as zonula occludens) controls the most apical cell-to-cell adhesion between neighboring epithelial and endothelial cells (Furuse (2010) and Bazzoni and Dejana (2004)). These TJ proteins include integral and transmembrane proteins, including the claudins, occludin, and junctional adhesion molecules (JAMs), which extend into the intercellular space and regulate the gate function, and cytoskeletal linker proteins, such as cingulin, ZO-1, ZO-2, and ZO-3, which anchor TJ-integral membrane proteins to the cell cytoskeleton (Heinemann and Schuetz (2019) and Heinemann and Schuetz (2019)). An uncontrolled flow of antigens across the intestinal epithelium and a malfunctioning intestinal barrier can tax the immune system of those who are vulnerable and alter the host–microbe balance, which can set off inflammatory processes in the gut or make mucosal infections more likely (Meddings (2008) and Yu et al. (2012)). A recent report showed that FTY720 therapy blunts the mucosal adaptive immune response, including the generation of Th1 cytokines, and impairs innate immunological responses, making mice more susceptible to *Citrobacter rodentium* infection (Murphy et al., 2012). However, the study did not assess the effect of FTY720 on the barrier function.

This study is designed and intended to investigate the effect of continuous dosing of FTY720 on the gut functional phenotype in a mouse model. We studied the TJ protein expression, gut permeability, and immune response in the gut of FTY720-treated mice compared with untreated control. Our results showed FTY720-treated mice exhibited downregulation of multiple TJ proteins, leading to an increase in gut permeability and inflammation, and these are associated with the inhibition of the AKT–mTOR signaling pathway. The mucosa of the mice shows indications of inflammation, including crypt hyperplasia, goblet cell loss, and immune cell infiltration at the crypt region. Our data clearly showed that continuous treatment with FTY720 increases the possibility of gut barrier disruption. To our knowledge, this is the first report indicating that FTY720 may jeopardize the critical barrier function and induce an immune response.

## Materials and methods

### Chemicals and reagents

Dulbecco’s modified Eagle’s medium (DMEM) and fetal bovine serum (FBS) were purchased from Gibco (Thermo Fisher Scientific). FTY720 (cat #SML0700) was purchased from Sigma-Aldrich. The cytokine detection ELISA kits were purchased from BD Biosciences (United States) and R&D Systems (United States). Primers were purchased from Integrated DNA Technologies. All the polyclonal antibodies for β-actin (sc-47778), occludin (ab216327), claudin-4 (ab53156), and claudin-2 (ab53032) and secondary anti-IgG (ab97080 and ab97023) antibodies were purchased from Santa Cruz Biotechnology and Abcam. For immunohistochemistry, an anti-MPO (PA5-16672, Invitrogen) antibody was used. Secondary IgG Alexa Fluor 488 (ab150081, Abcam) was used for TJ immunofluorescence. Rapamycin (cat #13346) was purchased from Cayman Chemical. The following antibodies were purchased and used for performing *in vitro* Western blot assay: p-Akt 1/2/3 (Ser 473) (cat #AF0016; Affinity), Akt (cat #GTX121937; GeneTex), P-mTOR (Ser 2,448) (D9C2) (cat #5536T; Cell Signaling Technology), and mTOR (7C10) (cat #2983S; Cell Signaling Technology). FACS antibodies used in immunofluorescence were as follows: FITC-CD3 (cat #100204; BioLegend), PerCP-CD19 (cat #115532; BioLegend), FITC-CD11b (cat #11-0112-85; eBioscience), and A700-Ly-6G (cat#127621; BioLegend).

### Mice and animal ethics

C57BL/6 mice (6 weeks old) were procured from the ICMR-NICED Animal Facility of the institute. All the protocol for the study was approved by the Institutional Animal Ethics Committee of ICMR-NICED, Kolkata, India (PRO/151/July 2018–June 2021). Experiments were carried out in accordance with the guidelines laid down by the committee for the purpose of the control and supervision of experiments on animals (CPCSEA), Ministry of Environment and Forests, Government of India, New Delhi, India.

### Treatment with FTY720

All mice were housed in cages containing straw bedding and held in pathogen-free facilities maintained at 24 °C with a 50% relative humidity and 12-h light:dark cycle. All mice had *ad libitum* access to standard rodent chow. The mice were divided into two groups (i.e., control and FTY720; n = 5/group). Mice in the FTY720 group were provided 3 mg/kg body weight (Choi et al., 2011) (Rau et al., 2011) of FTY720 orally for 14 days, whereas mice from the control group were provided normal saline. Food and water consumption was monitored throughout the experimental period. The body weights of the mice were also measured at regular time intervals. The mice were euthanized on day 15.

### Gut permeability assay

On the day of euthanasia, mice from both the control and treated groups were force-fed 44 mg/100 g body weight FITC-labeled 4-kDa dextran (FD-4; MW, 4,000 Da; Sigma-Aldrich; Merck KGaA) via gavage following fasting overnight (Liu et al. (2020), Chakraborty et al. (2023), and Kang et al. (2017)). After 4 h of FD-4 administration, blood was collected and centrifuged at 2,500 × g for 10 min at 4°C to collect the serum. The fluorescence intensity of FD-4 in the serum was determined using a multimode reader at an excitation and emission wavelength of 485 nm and 520 nm, respectively. Subsequently, a standard curve was generated using serial dilutions of FD-4 from 1,000 μg/mL to 0.

### Bacterial translocation

A 40 mg portion of mice liver was collected under aseptic conditions after the mice were euthanized. Liver samples were washed in 200 μL PBS containing gentamicin (50 μg/mL) and then homogenized in ice-cold PBS. A measure of 100 μL of each tissue homogenate was spread onto the nutrient agar and incubated at 37°C for 24 h. The number of bacterial colonies was counted based on the dilution factor and expressed as colony-forming unit (CFU). CFU/g of tissue denoted the amount of bacterial translocation.

### Real-time PCR

RNA was extracted using TRIzol Reagent (Invitrogen, Massachusetts, United States) from each colon sample and quantified using a NanoDrop 8000 Spectrophotometer (Thermo Fisher Scientific, United States). A measure of 0.5 µg of RNA was taken for reverse transcription with oligo dT primers, dNTPs, RNase inhibitor, and MuLV reverse transcriptase according to the kit manual (PrimeScript 1st strand cDNA Synthesis Kit, Takara Bio) to synthesize cDNA. Quantitative PCR was conducted using the SYBR green master mix (TB Green Premix Ex Taq-II) and ROX reagent in a real-time PCR system, and the ΔCt values were calculated to determine relative changes in target genes. The primer sets used were as follows: GAPDH (>NM_001411843.1) (5՛ACC​CAG​AAG​ACT​GTG​GAT​GG3՛) (Tm = 59.01); (5՛CAC​ATT​GGG​GTA​GGA​ACA​C3՛) (Tm = 55.49), product length = 170; occludin (>NM_001360538.1) (5՛TCA​CTT​TTC​CTG​CGG​TGA​CT3՛) (Tm = 59.53); (5՛GGG​AAC​GTG​GCC​GAT​ATA​ATG3՛) (Tm = 58.93), product length = 138; claudin-4 (>NM_009903.2) (5՛TCG​TGG​GTG​CTC​TGG​GGA​TGC​TT3՛) (Tm = 65.0); (5՛GCG​GAT​GAC​GTT​GTG​AGC​GGT​C3՛) (Tm = 62.8), product length = 170; claudin-2 (>NM_001410421.1) (5՛TAT​GTT​GGT​GCC​AGC​ATT​GT3՛) (Tm = 58.08); (5՛TCA​TGC​CCA​CCA​CAG​AGA​TA3՛) (Tm - 58.12), product length = 205; claudin-1 (>NM_016674.4) (5՛ATG​CAA​AGA​TGT​TTT​GCC​ACA​G3՛) (Tm = 58.60); 5՛TAC​AAA​TTC​CCA​TTG​CAG​CCC3՛) (Tm = 59.17), product length = 210; junctional adhesion molecule-A (JAM-A) (>NM_172647.2) (5՛CTG​ATC​TTT​GAC​CCC​GTG​AC3՛) (Tm = 58.27); (5՛ACC​AGA​CGC​CAA​AAA​TCA​AG3՛) (Tm = 56.9), product length = 187; mucin-2 (>NM_023566.4) (5ʹATG​TCC​TGA​CCA​AGA​GCG​AA3′) (Tm = 56.2); (5ʹGAT​TTG​AAG​GCC​ACC​ACG​TT3′) (Tm = 56.0), product length = 142; cathelicidin (>NM_009921.2) (5′GGC​AGC​TAC​CTG​AGC​AAT​GT3′) (Tm = 59.6); (5′CTG​TGC​ACC​AGG​CTC​GTT​A3′) (Tm = 59.8), product length = 122; and hepcidin (>NM_032541.2) (5ʹAGG​GCA​GAC​ATT​GCG​ATA​CC3ʹ) (Tm = 57.5); (5ʹGCA​ACA​GAT​ACC​ACA​CTG​GGA3ʹ) (Tm = 57), product length = 111.

### Cell culture

HT-29 cells, the human colorectal adenocarcinoma cells (kind gift from Dr. Sushmita Bhattacharya, ICMR-NICED), were cultured in DMEM with high glucose, supplemented with 10% FBS with 100 U/mL penicillin and 100 μg/mL streptomycin in humidified air/CO_2_ (5% CO_2_) at 37 °C within tissue culture flasks. Cell viability with FTY720 treatment at different doses was determined by 3-(4,5-dimethylthiazol-2-yl)-2,5-diphenyltetrazolium bromide. HT-29 cells were seeded with a density of 10^6^ cells/well and grouped into control, FTY720 (20 µM), and rapamycin (500 nM) groups, and incubated for 48 h. Thereafter, the cells were lysed with RIPA lysis buffer (5M NaCl, 0.5M EDTA, 1M Tris-HCl (pH 8.0), 0.5% Tween-20, 10% SDS, PMSF, and dH_2_O), followed by Western blotting, as described below.

### SDS PAGE and Western blotting

Colonic sections were washed with PBS and homogenized in RIPA lysis buffer. The total protein concentration of the sample was determined using Thermo Scientific™ Pierce™ BCA Protein Assay Kit (23225). A measure of 30–50 μg of each lysate was loaded into polyacrylamide gel (concentration of acrylamide was used according to the molecular weight of the target protein). After segregation, the proteins were transferred onto the PVDF membrane (IPVH00010, Millipore); 5% BSA in TBST was used as the blocking reagent. The membrane was incubated overnight with specific primary antibodies at 4°C. Then, the membrane was washed and incubated with AP/HRP-conjugated secondary antibody for 2 h at room temperature and detected using NBT BCIP substrate solution (Thermo Scientific)/chemiluminescent HRP substrate (Immobilon Western, Millipore). The HRP substrate-developed blots were visualized using the ChemiDoc Imaging System (Bio-Rad). Densitometric analysis of the protein bands was performed using ImageJ software.

### Immunofluorescence staining and imaging

Colon samples from each group were collected and fixed in 4% paraformaldehyde. Paraffin-embedded 5-μm-thick sections were generated. The sections were then de-paraffinized in xylene and rehydrated with graded ethanol, followed by distilled water. A measure of 1 mM EDTA buffer (pH 8.0) was used for antigen retrieval. The sections were then permeabilized with 0.1% sodium citrate and 0.5% Triton-X in TBST. The sections were incubated with blocking buffer (5% animal sera in TBST) at room temperature. The primary antibodies were added to the sections (1:200 for TJ antibodies and 1:50 for FACS antibodies) and incubated overnight at 4°C. The sections were then washed and incubated with the Alexa Fluor 488-conjugated secondary antibody for 2 h at room temperature, and counterstained with Hoechst 33342 (1 μg/mL) for 10 min at RT and then washed. The sections were mounted and visualized using a Carl Zeiss microscope equipped with a CCD camera, and the images were processed using ZEN software (Gumber et al., 2014). The fluorescence intensity of the target proteins was measured using ImageJ software.

### Histopathologic examination of the proximal colon

The proximal colon samples were fixed in 4% paraformaldehyde for 48 h at 4 °C. The fixed tissues were then dehydrated through graded alcohols and embedded in paraffin, and routine microtomy was then carried out to generate 5-mM sections. The sections, in turn, were stained with hematoxylin and eosin for later microscopic examination (Chakraborty and Bhaumik, 2020).

### Enzyme-linked immunosorbent assay for cytokine and chemokine profiling

To measure the inflammatory markers, that is, cytokines and chemokines, colon tissues were homogenized in PBS and centrifuged at 10,000 *g* for 1 min to collect the supernatants devoid of any tissue debris. The protein concentration of the lysates was quantified using the BCA Protein Assay Kit (Thermo Scientific™ Pierce™, United states). A measure of 50 μg lysate from each animal sample was used to perform ELISA for CXCL-1 (R&D Systems; DY453-05), CXCL-2 (R&D Systems; DY452-05), TNF-α (BD OptEIA; 555268), and IFN-γ (BD OptEIA; 555138) according to the manufacturer’s instructions.

### Statistical analysis

**FIGURE 1:**
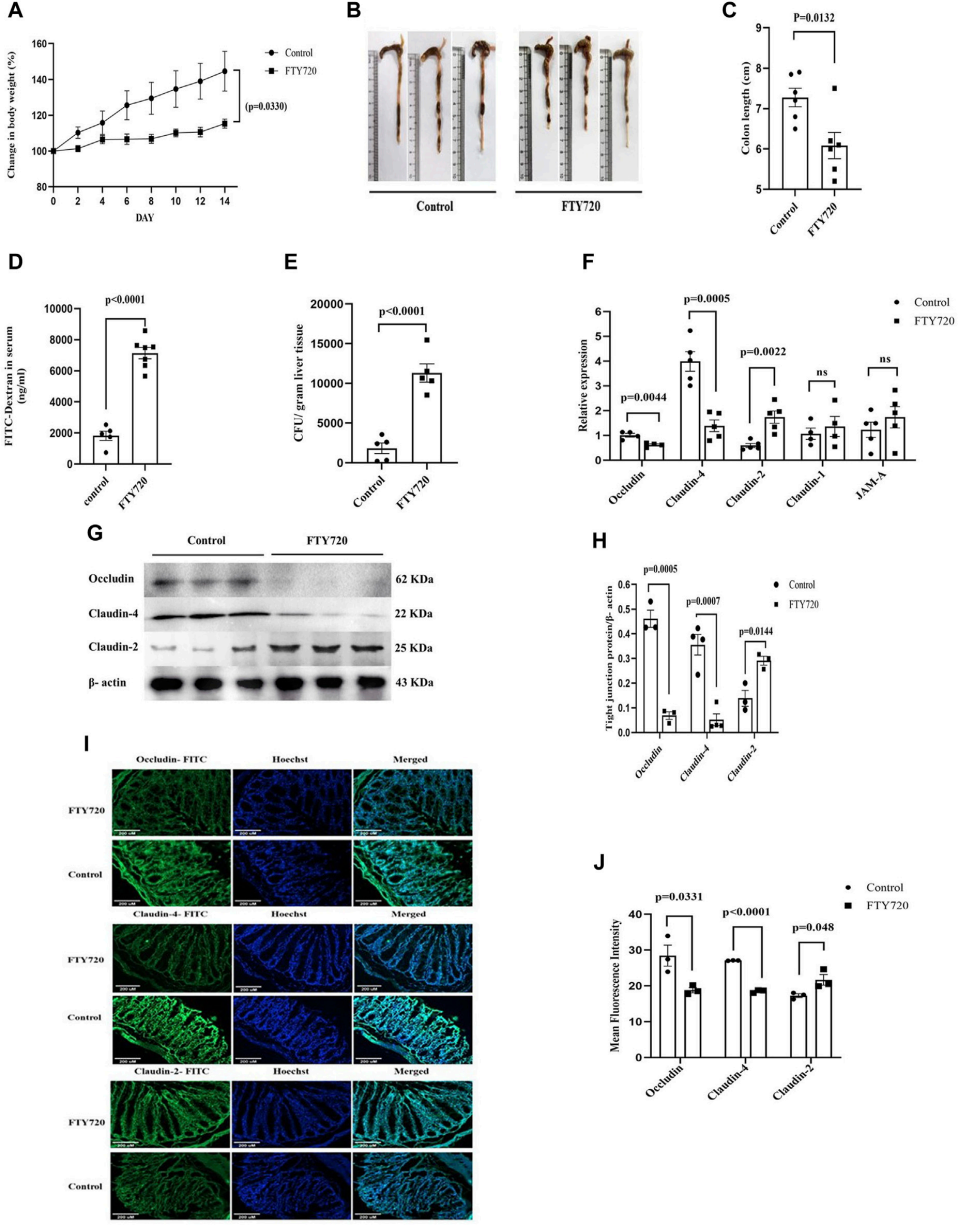
C57BL/6 mice were divided into control and FTY720 (3 mg/kg body weight) groups. After 14 days of continual dosing of FTY720, mice from both groups were euthanized. **(A)** The percentage change in body weight, **(B)** representative image of colon length, and **(C)** colon length of control mice and FTY720-treated mice were recorded. **(D)** After 4 h of oral administration of FITC dextran (44 mg/kg), there was a significant increase in FITC concentration in the sera among the FTY720 mice compared to that of the control mice. **(E)** The gut bacterial translocation in the liver was assessed by bacterial colony counting on a nutrient agar plate. **(F)** Real-time PCR data for tight junction-associated proteins: occludin, claudin-4, claudin-2, claudin-1, and JAM-A. **(G)** Western blot images of occludin, claudin-4, claudin-2, and, as internal control, β-actin from the colonic lysates of both control and FTY720 groups (full blot images are provided in Supplementary Figure S1). **(H)** Densitometric analysis of the Western blot images of occludin, claudin-4, and claudin-2. **(I)** Immunofluorescent images of colonic sections from control and FTY720 mice. Colon sections were stained with FITC-tagged (green) anti-occludin, anti-claudin-4, and anti-claudin-2 antibodies. Hoechst (blue) was used to stain the nucleus. Merged images represent the dual stains. **(J)** Densitometric analysis of the fluorescent intensity of the images. n ≥ 3 animals were taken per group; these data are represented as the mean ± SEM. *p* values were calculated with respect to the control.

**FIGURE 2:**
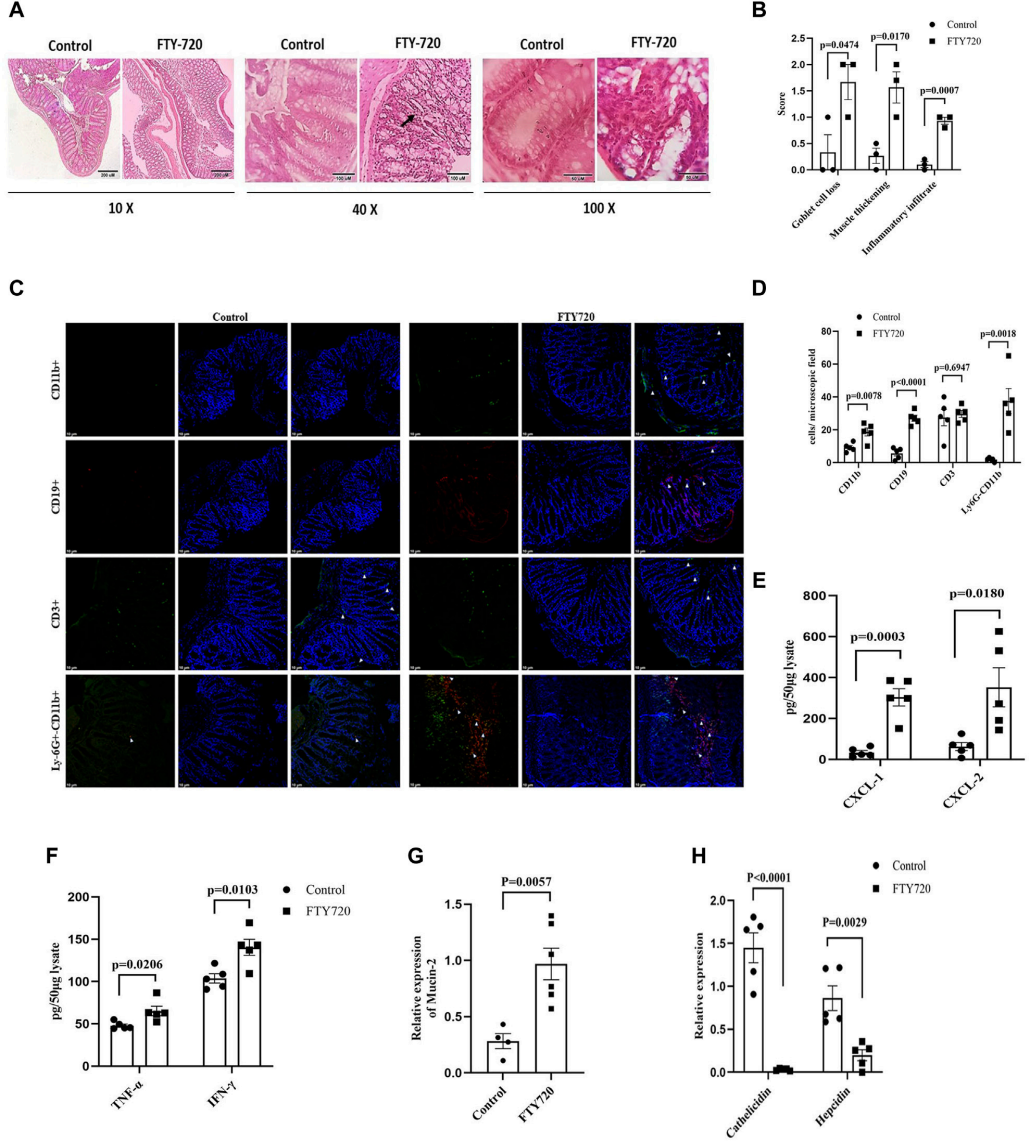
**(A)** Hematoxylin and eosin-stained colonic sections of mice from control and FTY720 groups (from left to right at 10 ×, 40 ×, and 100 × magnification, with the scale bar to the bottom right) for histological analysis. **(B)** Disease scores for goblet cell loss, muscle thickening, and infiltration of inflammatory cells. **(C)** Immunofluorescence of colon sections of FTY720 mice and control mice using FITC-conjugated anti-CD11b antibody, PerCP-conjugated anti-CD19 antibody, and FITC-conjugated anti-CD3 and A700-conjugated anti-Ly6G antibody. **(D)** The number of cells per microscopic field is represented. **(E)** CXCL-1 and CXCL-2 production in the gut tissue of FTY720 and control mice as measured by ELISA. **(F)** TNF-α and IFN-γ production in the gut tissue of FTY720 and control mice as measured by ELISA. **(G)** Real-time PCR analysis for mucus production regulating gene mucin-2. **(H)** Real-time PCR analysis for antimicrobial peptide cathelicidin and hepcidin from the colonic tissue of FTY720 and control mice. All the experiments were carried out with n ≥ 3 animals, and the graphs are presented as the mean ± SEM. *p*-values were calculated with respect to the control.

**FIGURE 3:**
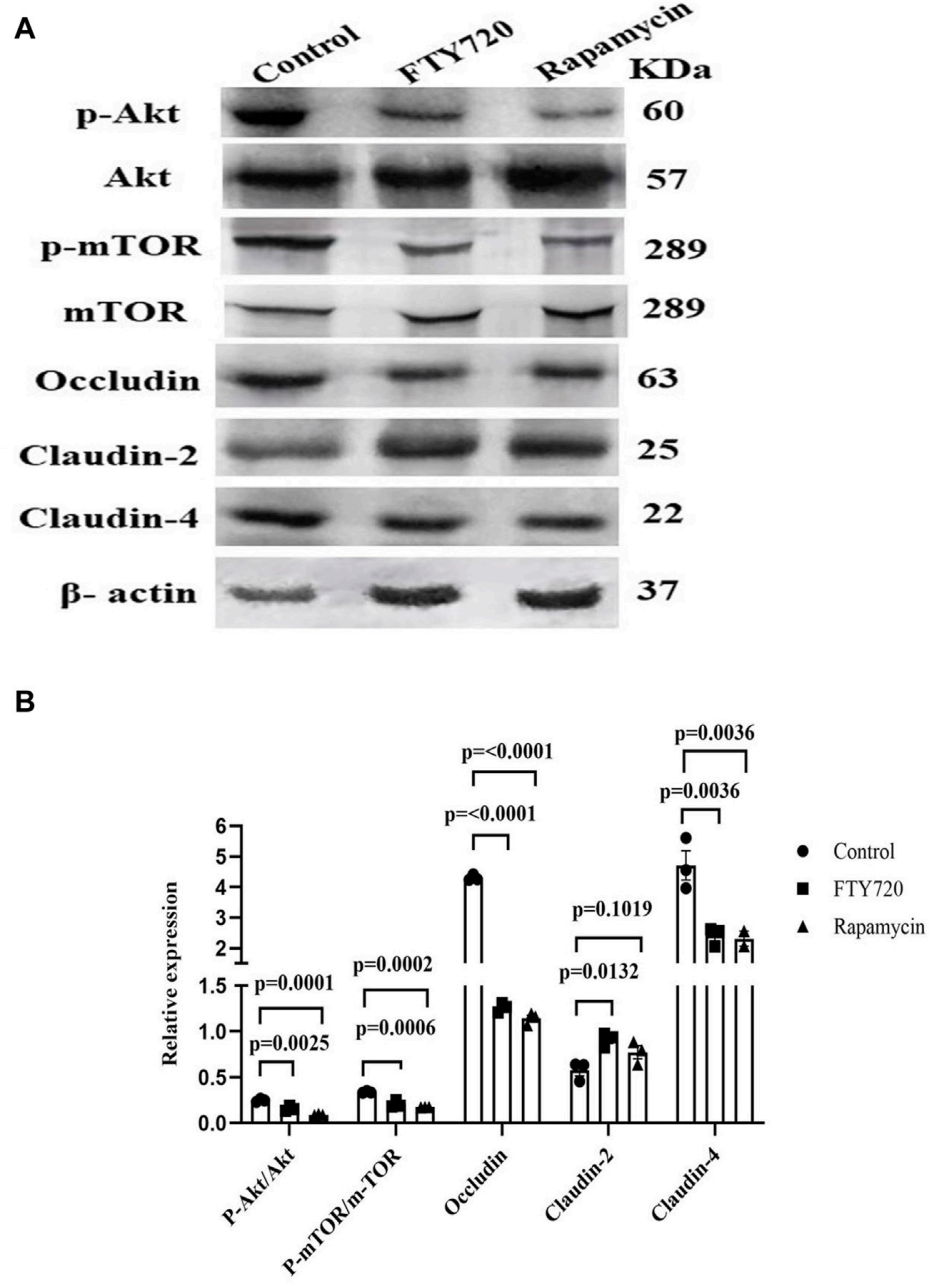
FTY720 inhibits Akt-mTOR to downregulate occludin and claudin-4 expression and upregulate claudin-2 expression in HT-29 cells. **(A)** HT-29 cells were treated with or without either FTY720 (20 µM) or rapamycin (500 nM) for 48 h. Thereafter, the cells were lysed, and Western blotting was conducted for p-AKT, AKT, p-mTOR, mTOR, occludin, claudin-2, claudin-4, and β-actin. **(B)** Densitometric analysis is represented for p-Akt/Akt, p-mTOR/mTOR, occludin/β-actin, claudin-2/β-actin, and claudin-4/β-actin (the full blot images are provided in Supplementary Figure S3).

All experiments were conducted in triplicate, and data are expressed as the mean ± SEM. In Figures 1, 2, Student’s *t*-test was used, and in Figure 3, one-way ANOVA was used using GraphPad Prism 8 software (GraphPad Software Inc., San Diego, CA, United States). Differences with *p* < 0.05 were considered significant.

## Results and discussion

Having strong immunosuppressive potential, FTY720 was approved as the first oral immunomodulatory drug for multiple sclerosis (Cohen et al. (2010) and Kappos et al. (2010)). This discourse attempts to shed light on the understanding of the effect of FTY720 on gut physiology. A multitude of evidence showed that S1P critically regulates the barrier function of mammalian cells (Kono et al., 2008) and that S1P manipulates the proteins that are involved in cell-to-cell adhesion (Greenspon et al., 2011). Adult C57BL/6 mice were divided into two groups: one group of mice received 3 mg/kg body weight FTY720 daily through oral gavage (FTY720-treated mice), and the other group was fed saline (control mice). FTY720 used in our study is completely water soluble; therefore, a vehicle control other than water was not required. The dose of FTY720 administration was previously used to treat multiple sclerosis in mice (Choi et al., 2011). All the animals received food and water *ad libitum*. Interestingly, the FTY720-treated mice did not exhibit any significant increase in body weight (Figure 1A), indicating stagnancy in growth. The gut length was significantly reduced in FTY720-treated mice compared to the control mice (Figures 1B, C). The function of the intestinal barrier, as investigated by FITC dextran permeability (Figure 1D), revealed that the intestinal permeability of FTY720-treated mice was 4.7 times higher (*p* < 0.0001) than that of the control mice.

The increase in barrier permeability was further confirmed by studying the translocation of bacteria in the liver (Figure 1E). It was observed that a significant number of bacteria were translocated to the liver in FTY720-treated mice compared to the control mice. The translocation of bacteria to the liver is an indication that FTY720 may predispose the individual to major complications and play a role in chronic liver disease. We looked at the group of proteins called occludin, classes of claudins (claudin-4, claudin-2, and claudin-1), and JAM-A that are associated with intestinal permeability (Figure 1F).

qPCR analysis of the expression of these proteins in the colon revealed a 2-fold reduction in occludin expression and a 2.9-fold decrease in claudin-4 expression, whereas a significant 2.92-fold increase in claudin-2 expression was observed in FTY720-treated mice compared to control mice, and claudin-1 and JAM-A expression remained unaltered. The results were validated by western blot analysis (Figures 1G, H) (full blot images are provided in Supplementary Figure S1) and immunofluorescence (Figures 1I, J). Consistent to our findings in qPCR, there was a decrease in occludin and claudin-4 expressions but an increase in claudin-2 expressions in FTY720-treated mice compared to control mice, as found in the western blot and immunofluorescence analyses. Tight junctions, which are intricate signaling hubs in a constantly changing environment that serve as an impermeable barrier to impede the free transit of solutes through the intercellular gap, make up the majority of proteins linked to gut permeability (Forster (2008) and Fasano (2012)). Paracellular permeability is largely regulated by tight junctional proteins; damage to their expression, assembly, or integrity causes an increase in epithelial permeability (Liang and Weber, 2014). Claudins form TJ strands with the cytoplasmic scaffold ZO and are essential for regulating the paracellular permeability (Furuse, 2010). In addition to claudins, TJs are the home of immunoglobulin superfamily proteins, including JAMs, and occludin, a tetra-spanning membrane protein (Furuse, 2010). The paracellular permeability is one of the main functions regulated by TJs. Occludin, with its coiled domain, functions to arrange the structural and functional components of TJs (Nusrat et al., 2000). As occludin is a crucial component of tight junctions, permeability increases when its expression or function is reduced. Increased intestinal permeability can lead to both local and systemic inflammatory pathways when luminal components translocate into the host (Mu et al., 2017). The largest interface the body has with the outside world is the gastrointestinal epithelium (Groschwitz and Hogan, 2009). The passage of luminal toxins, as well as the antigens, through the mucosa is selectively limited by the gut epithelium (Suzuki, 2013). The concept that mucosal inflammation results from a breach in the mucosal barrier is maintained by the physical positioning of the intestinal epithelial layer, which wedges between the mucosal surface and the luminal contents (Ahmad et al., 2017). Studies using TJ protein knockout mice showed that the gastrointestinal epithelium became inflamed (Ahmad et al., 2017) (Lu et al., 2013). Additional research confirms the critical significance of permeability, particularly with regard to its ability to support the overall function of the mucosal barrier in controlling mucosal immunological homeostasis (Ahmad et al., 2017). Histological analysis of the colon samples (Figure 2A) showed the infiltration of inflammatory cells in FTY720-treated mice compared to the control. The muscularis layer of the colon showed enlargement, and mucous-secreting goblet cells also showed hyperplasia in FTY720-treated mouse colon histological sections. The breakdown of the gut barrier and immune cell infiltration are closely related events (Lin et al., 2020). We observed a significant presence of CD19^+^ B cells, CD11b^+^ cells, and CD11b^+^ Ly6G^+^ cells (neutrophils) in the colon tissue of FTY720-treated mice compared to the control. However, there was no change in CD3^+^ T cells in the gut of FTY720-treated mice compared to the control mice (Figures 2C, D). The infiltration of CD19^+^ B cells, CD11b^+^ cells, and CD11b^+^ Ly6G^+^ neutrophils increased significantly in FTY720-treated mice compared to the control mice. FTY720, after being phosphorylated *in vivo*, binds to S1PRs (Wang et al., 1999; Van Brocklyn and Williams, 2012; Chen et al., 2017; Santacreu et al., 2019; Weigel et al., 2023) that are present on the lymphocyte surface and sequesters all the receptors except S1PR3, hence preventing lymphocyte egress from lymphoid organs. Conversely, phosphorylated FTY720 binds to S1PR3, which is present on the surface of neutrophils, macrophages, and B cells, and causes downward signaling (Sensken et al., 2008). Therefore, it is likely to find increased neutrophils, macrophages, and B cells but not T cells in the gut of FTY720-treated mice compared to the control mice. To ensure the inflammatory response in the gut due to FTY720 treatment, we measured the expression of CXCL-1 and CXCL-2, TNF-α, and IFN-γ (Figures 2E, F). Interestingly, there was a significant increase in CXCL-1 and CXCL-2, TNF-α, and IFN-γ levels in the colon of FTY720-treated mice compared to control. With increased CXCL-1 and CXCL-2, the neutrophil chemoattractants (Sawant et al., 2021) in FTY720 mice are also indicative of inflammation. It is unknown exactly how goblet cell hyperplasia occurs. A previous study reported that goblet cell hyperplasia is induced by IL-13, the major regulator of type-2-mediated inflammation, which speeds up inflammation (Huang et al., 2020).

S1PR2 has been linked to IL-13 (Zhao et al., 2018), which could possibly lead to goblet cell hyperplasia. Increased mucin-2 expression in FTY720 mice compared to the control is indicative of increased mucous production (Figure 2G). Previous research reported that colon mucin may be directly impacted by colonic inflammation (Kang et al., 2022). Subsequent investigation into the expression of antimicrobial genes, such as cathelicidin and hepcidin, revealed a notable decrease in FTY720-treated mice compared to the control (Figure 2H). It is thought to be crucial to colonic macrophage defense mechanisms against bacterial infection (Agier et al., 2015). Reduction in cathelicidin and hepcidin in the colon of FTY720-treated mice is indicative of the risk of opportunistic infection (McHugh et al. (2019), Scheenstra et al. (2020), and Ganz (2018)).

The expression of tight junction proteins is associated with the Akt–mTOR signaling pathway. In LS174T cells, human colon epithelial cells, it was shown that occludin expression is positively regulated by the PI3K–Akt-mTOR pathway (Mao et al., 2016). The Akt–mTOR pathway also activates autophagy, and the inhibition of autophagy contributes to the increase in claudin-2 expression (Zhang et al., 2017). Here, we show that FTY720 treatment to HT-29, a human colon carcinoma cell line, inhibits phosphorylation at Ser 473 of Akt and at Ser 2448 of mTOR, which is associated with a decrease in occludin and claudin-4 expression and an increase in claudin-2 expression compared to the control, as shown by Western blot analysis (Figures 3A, B) (full blot images are provided in Supplementary Figure S2). The dose of FTY720 treatment on HT-29 cells was determined previously by cell viability assay (Supplementary Figure S3). For Akt–mTOR phosphorylation, we used rapamycin, an inhibitor of mTOR, and found a similar decrease in the phosphorylation of Akt, mTOR, occludin, and claudin-4 and an increase in claudin-2 expression. The dose and time of rapamycin treatment were based on a previous report (Li et al., 2021). Therefore, it is tempting to speculate that FTY720 works through the inhibition of the mTOR signaling pathway to affect TJ protein expression. Further studies are warranted in this regard.

Among the latest findings, the most important one was that FTY720 impairs gut epithelial barrier regulation in mice. Moreover FTY720 caused a significantly increased intestinal barrier permeability and an elevated inflammatory response in the colon. These findings indicate that FTY720 causes deleterious effects in modulating the integrity of the intestinal barrier.

## Data Availability

The original contributions presented in the study are included in the article/Supplementary Material; further inquiries can be directed to the corresponding author.
