# Supplementary material for: The functional antagonist of sphingosine-1-phosphate, FTY720, impairs gut barrier function
Source: Front Pharmacol. 2024 Aug 19;15:1407228. doi: 10.3389/fphar.2024.1407228 (PMC11366638; doi:10.3389/fphar.2024.1407228)

Supplementary

Title: The functional antagonist of Sphingosine-1-Phosphate, FTY720 Impairs gut barrier function.

Sohini Sikdar[1], Debmalya Mitra[2], Oishika Das[1], Moumita Bhaumik[1*], Shanta Dutta[1]

Figure S1: Image of full blot that are represented in Figure- 1.G. The protein ladder used for the blot is for Occludin, Claudin-4, and Claudin-2 we used TaKaRa 3472 Broad range OG12 Colored Protein Ladder. For Beta Actin we used PageRuler Plus Prestained Protein Ladder, 10 to 250 KDa (26619, Thermo Scientific).

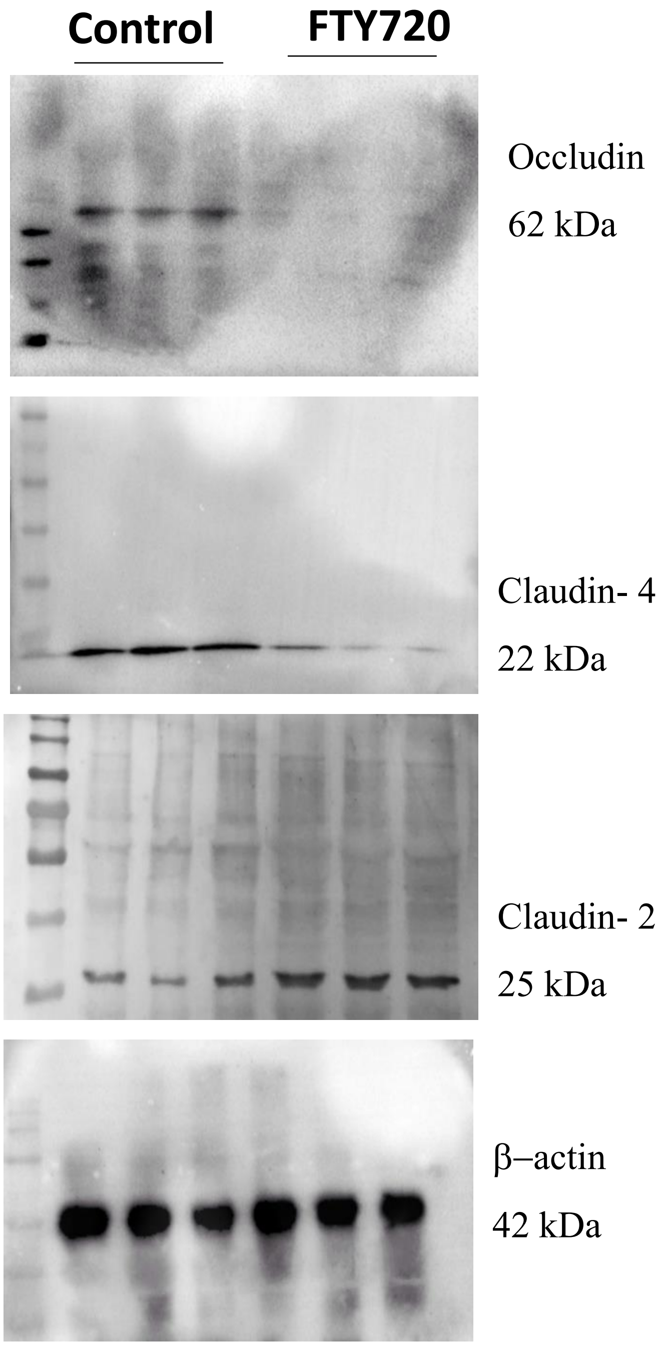

Figure- S2: Image of full blot that are represented in Figure- 3.A. 10% polyacrylamide gel was used for Akt, p-Akt, Occludin and b-actin, 8% polyacrylamide gel was used for mTOR and p-mTOR, 12% polyacrylamide gel was used for claudin- 2 and claudin- 4. The protein ladder used were TaKaRa 3472 Broad range OG12 Colored Protein Ladder and PageRuler Plus Prestained Protein Ladder, 10 to 250 KDa (26619, Thermo Scientific).

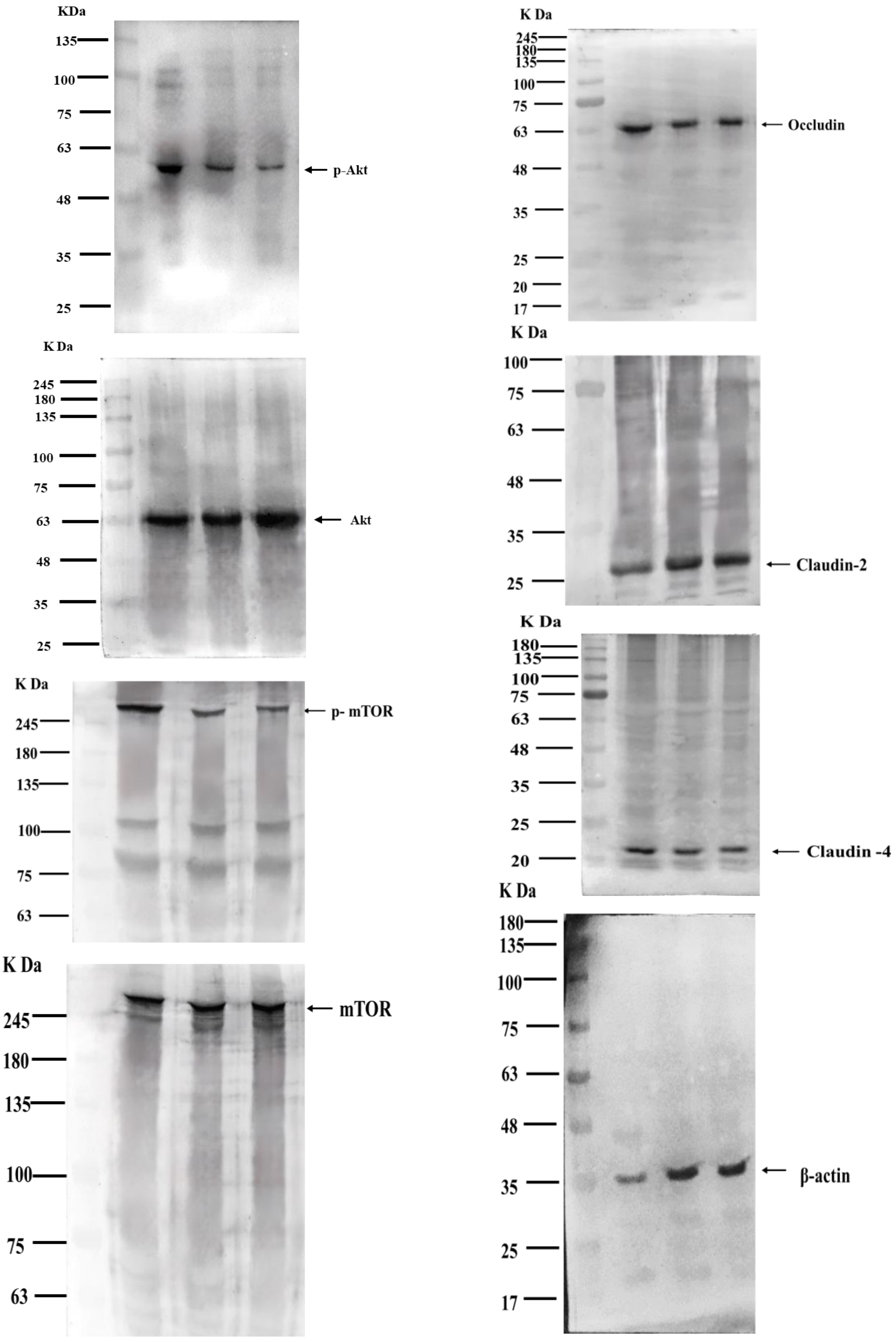

Figure- S3: HT-29 cells were treated with FTY720 at indicated doses for 48 hours. The cell viability was determined by MTT assay.

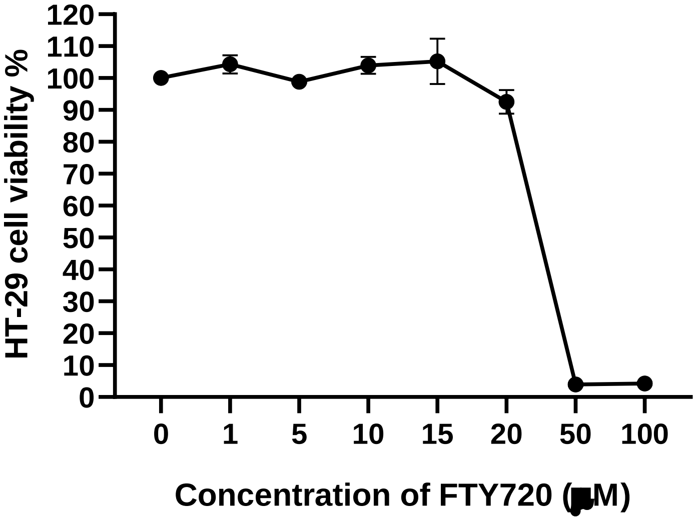

Supplement: Supplementary file 1 [file DataSheet1.pdf]
